# Supplementary material for: Deletion of wfs1 Impairs Oligodendrocyte Precursor Cells Dorsal Distribution and Myelination Through the wfs1-hmgcs1 Axis in Zebrafish
Source: Biology (Basel). 2026 Aug 16;15(16):1402. doi: 10.3390/biology15161402 (PMC13510151; doi:10.3390/biology15161402)
Supplement: Supplementary file 1 [file biology-15-01402-s001.zip › File S1-Transcriptome sequencing data and differentially expressed genes (DEGs) in wfs1-- zebrafish at 5 dpf/1.GO/wfs1KOvsWT/GSEA/WT/MUSCLE_CELL_PROLIFERATION/MUSCLE_CELL_PROLIFERATION(GO_0033002).html]

Details for gene set MUSCLE\_CELL\_PROLIFERATION(GO:0033002)[GSEA]


|  || Dataset | deg.sample |
| Phenotype | sample.cls |
| Upregulated in class | WT |
| GeneSet | MUSCLE\_CELL\_PROLIFERATION(GO:0033002) |
| Enrichment Score (ES) | -0.4873948 |
| Normalized Enrichment Score (NES) | -1.6980363 |
| Nominal p-value | 0.0 |
| FDR q-value | 0.32927772 |
| FWER p-Value | 0.927 |
Table: GSEA Results Summary

  

Fig 1: Enrichment plot: MUSCLE\_CELL\_PROLIFERATION(GO:0033002)
  
Profile of the Running ES Score & Positions of GeneSet Members on the Rank Ordered List

  

| PROBE | DESCRIPTION   (from dataset) | GENE SYMBOL | GENE\_TITLE | RANK IN GENE LIST | RANK METRIC SCORE | RUNNING ES | CORE ENRICHMENT || 1 | 796720 | si:ch211-263k4.2 |  |  | 4504 | 0.744 | -0.0856 | No |
| 2 | 567448 | mfn2 |  |  | 5011 | 0.680 | -0.0505 | No |
| 3 | 768121 | foxo1a |  |  | 6230 | 0.565 | -0.0464 | No |
| 4 | 560653 | npr1a |  |  | 8865 | 0.377 | -0.1008 | No |
| 5 | 561406 | ptgir |  |  | 9812 | 0.315 | -0.1069 | No |
| 6 | 404627 | six1b |  |  | 11578 | 0.212 | -0.1464 | No |
| 7 | 494168 | six1a |  |  | 11745 | 0.204 | -0.1363 | No |
| 8 | 100334610 | nppb |  |  | 13209 | 0.130 | -0.1725 | No |
| 9 | 30694 | nkx2.7 |  |  | 14186 | 0.083 | -0.1969 | No |
| 10 | 562912 | ltbp3 |  |  | 14215 | 0.082 | -0.1916 | No |
| 11 | 100331745 | npr2 |  |  | 14217 | 0.081 | -0.1856 | No |
| 12 | 321587 | naa35 |  |  | 15503 | 0.022 | -0.2242 | No |
| 13 | 692305 | ndrg4 |  |  | 18960 | -0.120 | -0.3237 | No |
| 14 | 334956 | larp7 |  |  | 21299 | -0.230 | -0.3798 | No |
| 15 | 795653 | vip |  |  | 21405 | -0.236 | -0.3654 | No |
| 16 | 323695 | cav1 |  |  | 21810 | -0.257 | -0.3588 | No |
| 17 | 58126 | runx1 |  |  | 22962 | -0.324 | -0.3706 | No |
| 18 | 353295 | smarca4a |  |  | 23865 | -0.384 | -0.3702 | No |
| 19 | 321442 | nppa |  |  | 27602 | -0.685 | -0.4360 | Yes |
| 20 | 58146 | hey2 |  |  | 27615 | -0.689 | -0.3848 | Yes |
| 21 | 30696 | nkx2.5 |  |  | 28767 | -0.871 | -0.3556 | Yes |
| 22 | 57936 | tbx20 |  |  | 28830 | -0.884 | -0.2913 | Yes |
| 23 | 30592 | myl7 |  |  | 29608 | -1.098 | -0.2334 | Yes |
| 24 | 569395 | npr3 |  |  | 30132 | -1.305 | -0.1520 | Yes |
| 25 | 406432 | akirin1 |  |  | 31446 | -2.764 | 0.0139 | Yes |
Table: GSEA details
[plain text format]

  

Fig 2: MUSCLE\_CELL\_PROLIFERATION(GO:0033002)
  
Blue-Pink O' Gram in the Space of the Analyzed GeneSet

  

Fig 3: MUSCLE\_CELL\_PROLIFERATION(GO:0033002): Random ES distribution
  
Gene set null distribution of ES for
 **MUSCLE\_CELL\_PROLIFERATION(GO:0033002)**
